# Novel MHC-Independent αβTCRs Specific for CD48, CD102, and CD155 Self-Proteins and Their Selection in the Thymus

**DOI:** 10.3389/fimmu.2020.01216

**Published:** 2020-06-16

**Authors:** François Van Laethem, Ingrid Saba, Jinghua Lu, Abhisek Bhattacharya, Xuguang Tai, Terry I. Guinter, Britta Engelhardt, Amala Alag, Mirelle Rojano, Jennifer M. Ashe, Ken-ichi Hanada, James C. Yang, Peter D. Sun, Alfred Singer

**Affiliations:** ^1^Experimental Immunology Branch, National Cancer Institute, National Institutes of Health, Rockville, MD, United States; ^2^Structural Immunology Section, Laboratory of Immunogenetics, National Institute of Allergy and Infectious Diseases, Rockville, MD, United States; ^3^Theodor Kocher Institute, Faculty of Bern, Universität Bern, Bern, Switzerland; ^4^Surgery Branch, National Cancer Institute, National Institutes of Health, Rockville, MD, United States

**Keywords:** MHC-independent, thymic selection, MHC restriction, tolerance, coreceptors

## Abstract

MHC-independent αβTCRs (TCRs) recognize conformational epitopes on native self-proteins and arise in mice lacking both MHC and CD4/CD8 coreceptor proteins. Although naturally generated in the thymus, these TCRs resemble re-engineered therapeutic chimeric antigen receptor (CAR) T cells in their specificity for MHC-independent ligands. Here we identify naturally arising MHC-independent TCRs reactive to three native self-proteins (CD48, CD102, and CD155) involved in cell adhesion. We report that naturally arising MHC-independent TCRs require high affinity TCR-ligand engagements in the thymus to signal positive selection and that high affinity positive selection generates a peripheral TCR repertoire with limited diversity and increased self-reactivity. We conclude that the affinity of TCR-ligand engagements required to signal positive selection in the thymus inversely determines the diversity and self-tolerance of the mature TCR repertoire that is selected.

## Introduction

The ligand recognition specificity of the αβT cell receptor (TCR) repertoire is established during T cell differentiation in the thymus. The recombination activating genes Rag1 and Rag2 induce random TCR gene re-arrangements in immature thymocytes and those with productively rearranged TCRα and TCRβ genes express αβTCR protein complexes on their cell surfaces (1). These randomly generated surface αβTCR complexes constitute the pre-selection TCR repertoire and display a huge diversity of potential ligand recognition specificities from which the mature TCR repertoire is selected in the thymus (2). During thymic selection, many pre-selection TCR specificities are lost because they fail to engage an intra-thymic ligand and consequently fail to signal thymocyte survival and maturation. Only immature thymocytes whose TCRs successfully engage an intra-thymic ligand generate TCR-mediated survival signals and differentiate into mature T cells, events referred to as positive selection (3, 4).

The mature TCR repertoire that is positively selected in normal mice is specific for linear antigenic peptides bound to Major Histocompatibility Complex (MHC)-encoded molecules, a recognition feature known as “MHC restriction” (5–7). In contrast the pre-selection TCR repertoire from which the mature repertoire is selected includes both MHC-restricted TCRs specific for peptide-MHC (pMHC) ligands as well as MHC-independent TCRs specific for conformational epitopes on native protein ligands (8). It has been proposed that the thymus positively selects an MHC-restricted TCR repertoire because MHC-restricted TCRs engage intra-thymic peptide-MHC (pMHC) ligands together with CD4/CD8 coreceptors whose cytosolic tails are associated with p56Lck (Lck) protein tyrosine kinase molecules that initiate TCR-mediated positive selection signaling; in contrast, MHC-independent TCRs bind to intra-thymic protein ligands independently of CD4/CD8 coreceptors and so cannot access coreceptor-associated Lck to signal positive selection (9). However, in CD4/CD8 coreceptor-deficient mice, Lck in immature thymocytes is not sequestered by coreceptors and so is available to transduce signals from all ligand-engaged TCRs, including TCRs specific for MHC-independent ligands. Consequently, in coreceptor-deficient mice that additionally lack MHC [so-called QuadKO mice (10)], MHC-independent TCRs signal immature thymocytes to undergo positive selection and to differentiate into mature T cells expressing only an MHC-independent TCR repertoire. Thus, unlike the mature TCR repertoire in coreceptor-expressing mice which is MHC-restricted and specific for pMHC ligands, the mature TCR repertoire in coreceptor- and MHC-deficient QuadKO mice is MHC-independent and specific for conformational epitopes on native protein ligands (9, 11, 12).

Relatively little is known about the specificity and thymic selection requirements of MHC-independent TCRs because only two individual TCRs from QuadKO mice (named A11 and B12A) have been identified and characterized in detail (11, 12). Curiously, these two TCRs bind to different conformational epitopes on the same self-protein which is CD155 (the murine analog of the human polio virus receptor), and they do so with ~10-fold higher affinity than that with which conventional MHC-restricted TCRs bind to foreign antigenic pMHC ligands (11). Most surprising is that both CD155-specific TCRs require intra-thymic CD155 to signal positive selection in the thymus (12) which contrasts starkly with conventionally MHC-restricted TCRs that only require very low affinity ligand engagements to signal positive selection (13–15). However, it is not certain if high affinity ligand engagements are required for positive selection of other MHC-independent TCRs or if such a requirement is unique to the two CD155-specific TCRs A11 and B12A.

We undertook the present study to determine if MHC-independent TCRs required high affinity TCR-ligand engagements to signal positive selection and, if so, to determine the consequences of high affinity thymic selection on the mature TCR repertoire. We now identify and characterize MHC-independent TCRs that recognize new MHC-independent ligands: TCR-38 is specific for CD48 and TCR-146 is specific for ICAM-2 (CD102). Like CD155, CD48, and CD102 also function as low affinity ligands for cell adhesion receptors. We focused on TCR-146 which binds exclusively to ICAM-2 (CD102) and found that it bound with high 1.6 μM affinity independently of LFA-1. In the thymus, TCR-146 strictly requires ICAM-2 to signal positive selection, indicating that MHC-independent TCRs generally require high affinity ligand engagements to signal positive selection which is very different from conventional MHC-restricted TCRs. Importantly, we discovered that the requirement for high affinity ligand engagements in the thymus selects a peripheral MHC-independent TCR repertoire with markedly limited receptor diversity and increased self-reactivity. In contrast, dependence on CD4/CD8 coreceptors allows conventional MHC-restricted TCRs to signal positive selection with very low affinity ligand engagements which generates a peripheral TCR repertoire that is both highly diverse and self-tolerant. We conclude that the affinity of TCR-ligand engagements that signal positive selection in the thymus profoundly affects the diversity and self-reactivity of the selected TCR repertoire.

## Results

### Identification of Novel MHC-Independent αβTCRs

The present study was undertaken to enhance understanding of MHC-independent TCR ligand recognition, positive selection, and repertoire generation. Because MHC-independent TCRs are positively selected in Quad^KO^ (*H2-Ab*^−/−^*B2m*^−/−^*Cd4*^−/−^*Cd8*^−/−^) mice, we began by generating T-hybridoma cell lines from Quad^KO^Bcl-2^Tg^ (QB) LNT cells that express the pro-survival Bcl-2^Tg^ to minimize loss of TCR specificities from *in vivo* signaled cell death (16).

**Figure 1:**
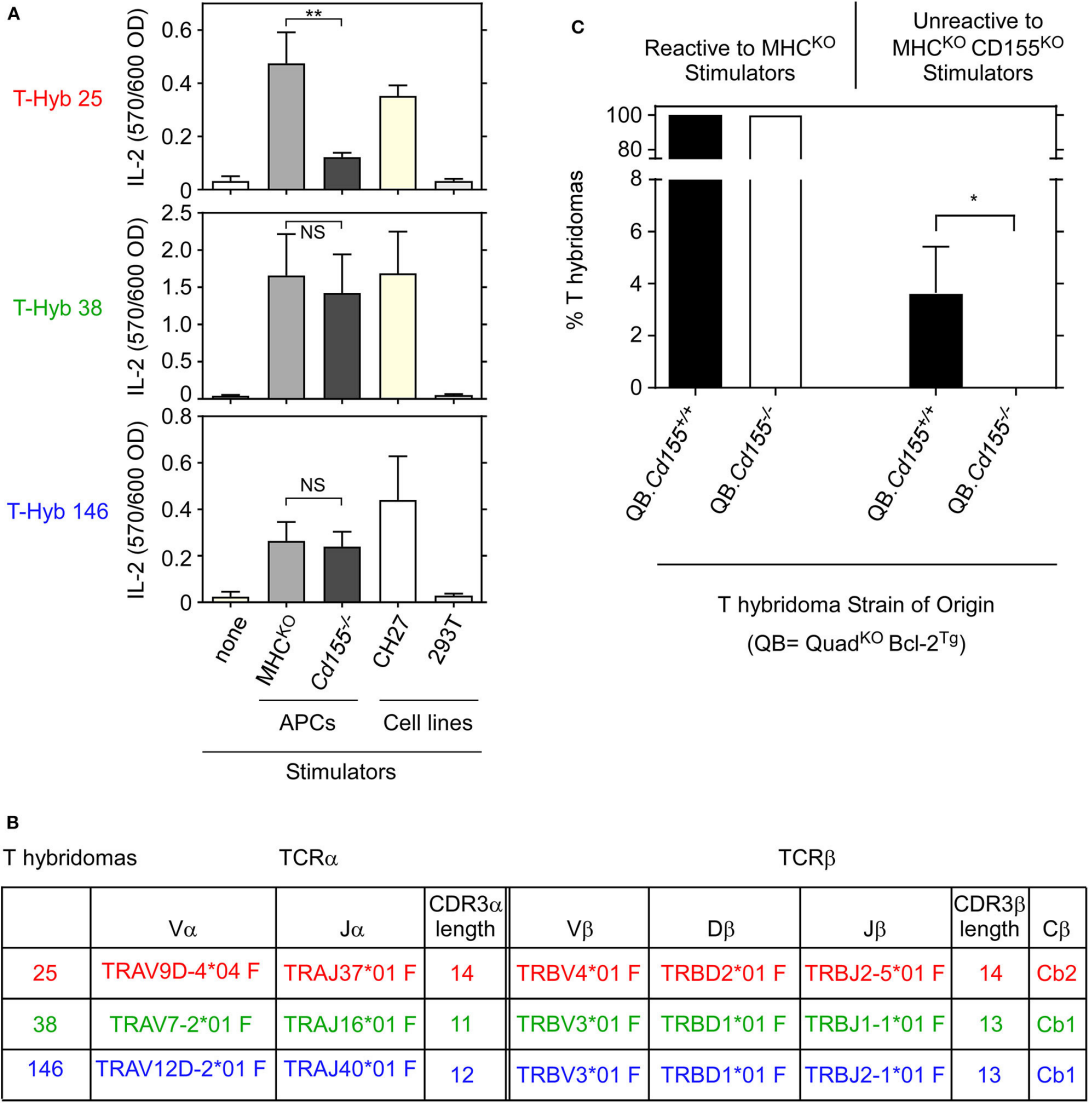
Reactivity of MHC-independent T-hybridomas from Quad^KO^ mice. **(A)** Reactivity of T hybridomas 25, 38, and 146 generated from Quad^KO^Bcl2^Tg^ (QB) mice. T-hybridoma cells (1 × 10^5^) were cocultured with stimulator cells (2 × 10^5^) for 16 hr and assayed for IL-2 production by ELISA. Each point represents the mean ± SEM of triplicate cultures. Data are representative of three independent experiments. **(B)** Characterization of αβTCRs from T-hybridomas 25, 38, and 146. T-hyb 25 contained Vα3 and Vβ10 TCR chains; T-hyb 38 contained Vα1 and Vβ16 TCR chains; and T-hyb 146 contained Vα8 and Vβ16 TCR chains. **(C)** CD155-specific T hybridomas cannot be generated with LNT cells from CD155-deficient mice. Four independent fusions were performed in parallel with LNT cells from Quad^KO^Bcl-2^Tg^ (QB) and QB. *Cd155*^−/−^ mice, with each fusion from each strain generating ~100 T-hybridomas. T-hybridomas reactive to platebound anti-TCR+anti-CD28 mAbs were then assessed for reactivity against MHC^KO^ and MHC^KO^CD155^KO^ spleen stimulator cells. One hundred percent of such T-hybridomas recognized an MHC-independent surface ligand as they reacted against MHC^KO^ stimulators, while some frequency of T-hybridomas in each fusion were CD155-specific as they were unreactive against MHC^KO^CD155^KO^ stimulators. **p < 0.01; *p < 0.5; NS, not significant.

We generated T-hybridomas from QB LNT cells that had been stimulated with platebound anti-TCRβ/anti-CD28 antibodies and screened them for recognition of MHC-independent ligands expressed on MHC^KO^ antigen presenting cells (APCs) (Figure 1A). Three T-hybridomas (T-hyb 25, T-hyb 38, and T-hyb 146) were selected for further study. T-hyb 25 reacted against MHC^KO^ APC stimulators but not CD155^KO^ APC stimulators, indicating that its MHC-independent ligand was CD155, whereas the other two T-hybridomas (T-hyb 38 and T-hyb 146) reacted against both MHC^KO^ and CD155^KO^ spleen APC stimulators indicating that their MHC-independent ligands were molecules other than CD155 (Figure 1A). TCR sequencing of the selected T-hybridoma lines revealed that each TCR expressed a single TCRα and a single TCRβ chain, so that TCR-25 was Vα3 Vβ10 (TRAV9D TRBV4); TCR-38 was Vα1 Vβ16 (TRAV7 TRBV3); and TCR-146 was Vα8 Vβ16 (TRAV12D TRBV3) (Figure 1B). Complete amino-acid sequences of these TCRs are displayed in Figure S1.

### *In vivo* Ligand Expression Is Required for Generation of Ligand-Specific T Cells

Because CD155-specific T-hybridomas appear frequently in BW5147 fusions with QB LNT cells (11, 12), we could ask if generation of CD155-specific T cells required *in vivo* CD155 expression in QB mice. To answer this question, we performed parallel T-hybridoma fusions with LNT cells from CD155-sufficient (CD155^+/+^) and CD155-deficient (CD155^−/−^) QB LNT cells (Figure 1C), generating approximately 400 individual T-hybridomas in four independent fusions with LNT cells from each mouse strain. We found that all T-hybridomas from CD155^+/+^ and CD155^−/−^ QB mice expressed MHC-independent TCRs that reacted against MHC^KO^ spleen APC stimulators (Figure 1C left), and that a subset of these expressed CD155-specific TCRs that failed to react against MHC^KO^CD155^−/−^ APCs (Figure 1C right). Strikingly, ~4% of T-hybridomas from CD155-sufficient LNT cells were CD155-reactive, whereas none (0%) of the T-hybridomas from CD155-deficient LNT cells were CD155-reactive (*p* < 0.05) (Figure 1C right). Thus CD155-specific TCRs are not generated with LNT cells from CD155-deficient mice, indicating that *in vivo* CD155 expression is required for positive selection of CD155-specific MHC-independent TCRs.

### Identification of CD102 and CD48 as MHC-Independent TCR Ligands

We then wished to determine if the requirement for *in vivo* ligand expression is limited only to TCRs specific for CD155 or if it extends to TCRs specific for other MHC-independent ligands as well. However, no other MHC-independent TCR ligands have yet been identified. Consequently, we embarked on identifying the MHC-independent ligands recognized by the three T-hybridomas that we had selected to study. We first verified that all three T-hybridomas reacted against ligands expressed on the murine CH27 B cell line but did not react to ligands expressed on the human 293T cell line (Figure 1A). We then transfected a cDNA library made from stimulatory CH27 cells into non-stimulatory human 293T cells and performed limiting dilution cDNA expression cloning (11) (Figure S2). In this way, we ultimately identified three cDNA clones whose transfection into 293T cells converted them into stimulatory cells for each T-hybridoma. We determined that the transfected cDNA stimulating T-hyb 146 encoded ICAM-2 (CD102); the transfected cDNA stimulating T-hyb 38 encoded CD48; and the transfected cDNA stimulating T-hyb 25 encoded CD155.

**Figure 2:**
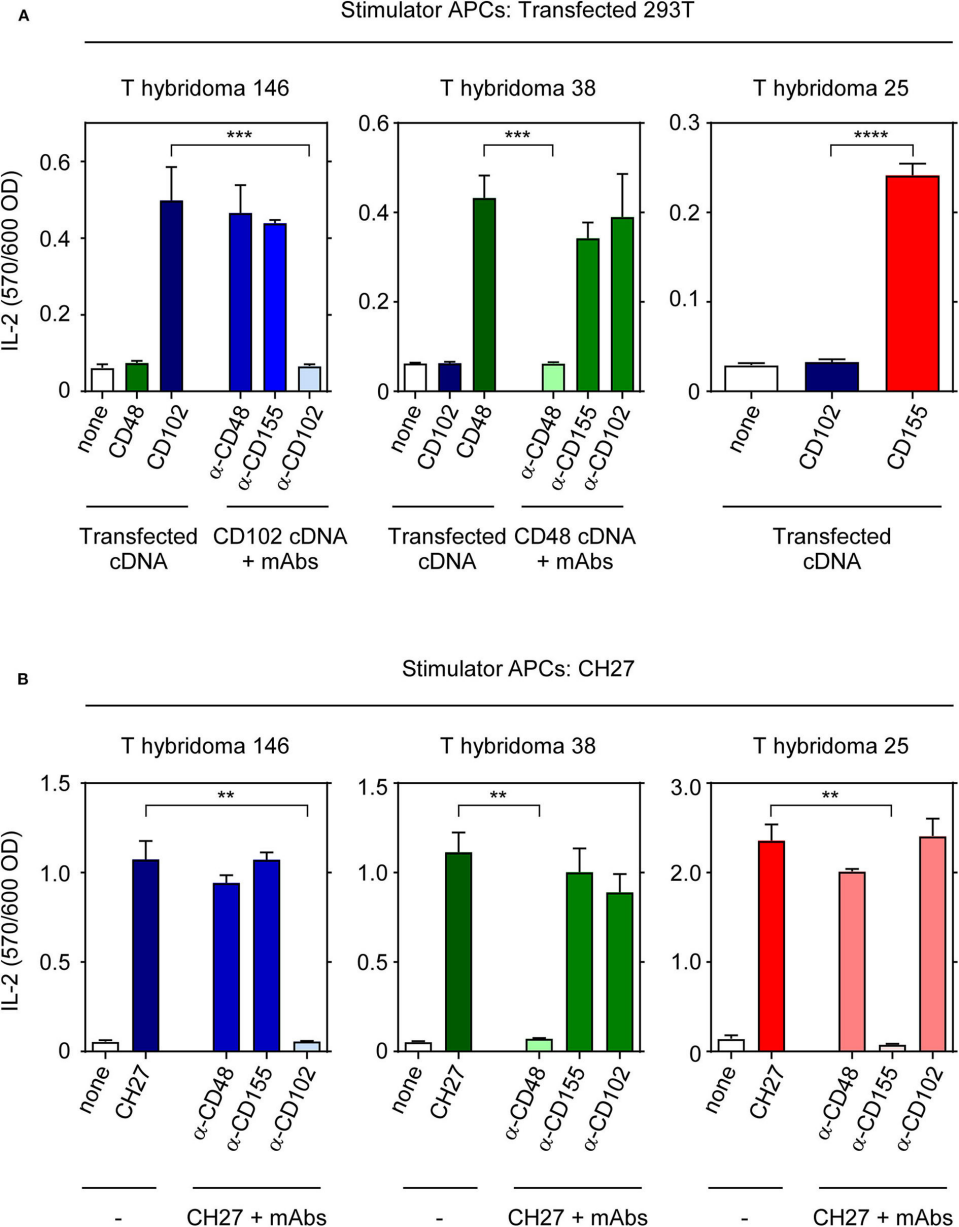
Identification of MHC-independent ligands for TCRs 25, 38, and 146. **(A)** IL-2 production by T hybridomas 25, 38, and 146 after 16 h stimulation with 1 × 10^5^ 293T cells transfected with the indicated cDNAs. Where indicated, blocking mAbs (10 μg/ml) were added at the beginning of cell culture. **(B)** IL-2 production by hybridomas after 16 h stimulation with 1 × 10^4^ CH27 cells in the presence or absence of blocking mAbs. Data are representative of three independent experiments. ****p < 0.0001; ***p < 0.001; **p < 0.01.

To verify their ligand specificities, we stimulated each T-hybridoma with human 293T cells that had been transfected with known cDNAs encoding CD102, CD48, and CD155 (Figure 2A). Indeed, T-hyb 146 reacted only against 293T cells transfected with CD102 cDNA and its reactivity was blocked only by anti-CD102 monoclonal antibody (mAb); T-hyb 38 reacted only against 293T cells transfected with CD48 cDNA and its reactivity was blocked only by anti-CD48 mAb; and T-hyb 25 reacted only against 293T cells transfected with murine CD155 (Figure 2A). For T-hyb 25, we performed domain-swapping between human and murine CD155 to map the CD155 epitope recognized by TCR-25 (Figure S3) and found that stimulation of T-hyb 25 requires that all 3 external CD155 domains be murine (not human) sequences, indicating that TCR-25 recognizes a novel epitope formed by all three external domains of murine CD155 that has not been previously described (11) (Figure S3). Thus, TCR-146 recognizes ICAM-2 (CD102), TCR-38 recognizes CD48, and TCR-25 recognizes a unique CD155 epitope.

To determine if there might be additional stimulatory ligands for each T-hybridoma, we assessed the ability of ligand-specific mAbs to block each T-hybridoma's reactivity against murine CH27 stimulator B-cells (Figure 2B). While conventionally MHC-restricted TCR responses are never blocked by anti-ligand antibodies, MHC-independent TCR responses are blocked by anti-ligand mAbs (11). Interestingly, we found that the reactivity of T-hyb 146 is blocked by anti-CD102; that of T-hyb 38 is blocked by anti-CD48; and that of T-hyb 25 is blocked by anti-CD155 (Figure 2B). Thus, we have identified novel MHC-independent TCRs with specificity for two novel ligands (CD102 and CD48) and a novel epitope on CD155.

### MHC-Independent TCRs Recognize Native Ligands Without Antigen Processing

**Figure 3:**
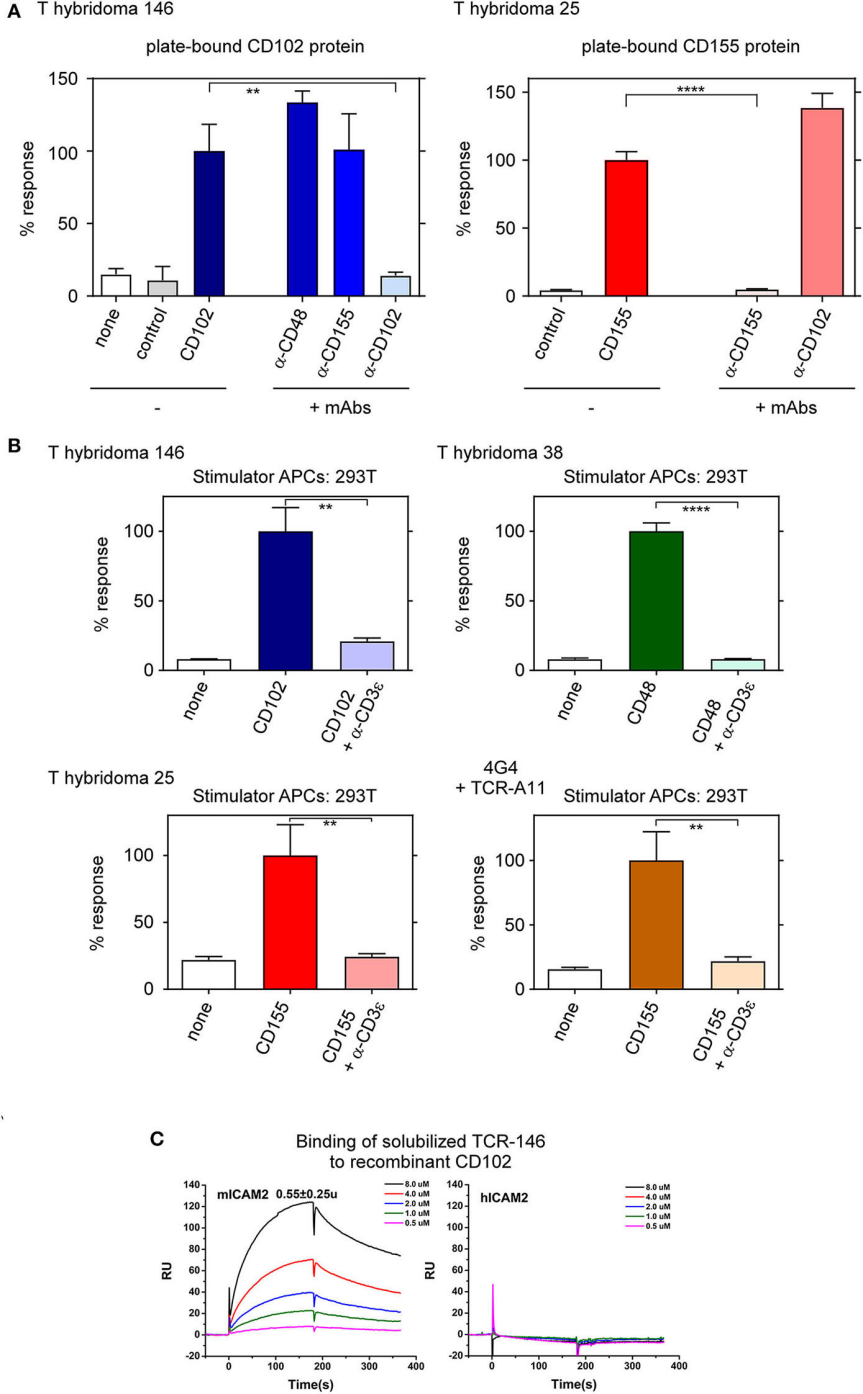
T-hybridomas recognize and react to recombinant protein ligands immobilized on plastic. **(A)** Responses of T-hybridomas (1 × 10^5^) to immobilized (10 μg/ml) protein in the presence or absence of blocking antibodies. Responses of T-hyb 146 and T-hyb 25 against CD102 and CD155, respectively, were each set at 100%. Each data point represents the mean ± SEM of triplicate experiments. **(B)** Responses of T-hyb 146, 38, 25, and TCR-transduced 4G4 cells (TCR A11) against 293T cells transfected with their respective ligand-encoding cDNA in the presence or absence of blocking anti-CD3ε mAb (clone 7D6, 10 μg/ml). **(C)** Binding of soluble TCR 146 to recombinant CD102. Surface plasmon resonance (SPR) measurements of binding between solubilized TCR 146 αβTCRs heterodimers and immobilized recombinant murine CD102 (left panel) or human CD102 (right panel). The analytes consisted of serial dilutions of soluble αβTCR heterodimers ranging from 0.5 to 8 μM. The dissociation constants were obtained by kinetic curve fitting via BIAevaluation. Data are representative of two independent experiments. ****p < 0.0001; **p < 0.01.

Because antibodies bind to conformational epitopes on native proteins, blockade of T-hybridoma reactivity by ligand-specific antibodies suggests that their MHC-independent TCRs recognize native protein ligands. Consequently, we might be able to stimulate these T-hybridomas with recombinant proteins immobilized on plastic in the absence of APCs. Indeed T-hyb 146 specifically responds to plate-bound recombinant CD102 protein but not to recombinant CD155 (control) protein, while T-hyb 25 reacts to plate-bound CD155 protein but not CD102 protein (Figure 3A). Moreover, the response of each T-hybridoma to its plate-bound protein ligand is blocked by mAb specific for that protein ligand (Figure 3A).

Interestingly, the stimulatory ligand for each of these T-hybridomas (i.e. CD102, CD48, and CD155) is an adhesion molecule that can also bind with low affinity to counter-receptors on lymphocytes, as ICAM-2 (CD102) binds to LFA-1 and Mac-1 (17, 18); CD48 binds to CD2 and 2B4 (19); and CD155 binds to CD226, CD96, TIGIT, vitronectin, and CD113 (20, 21). Even though adhesive interactions are low affinity, we wanted to verify that the specific reactivity of each T-hybridoma is signaled by its clonotypic TCR rather than by its adhesive counter-receptor. To do so, we stimulated T-hybridomas with 293T cell transfectants and assessed the ability of soluble anti-CD3ε mAb to inhibit the transduction of ligand-specific responses (Figure 3B). As a positive control for anti-CD3ε inhibition of TCR responses, we included a responder cell line (4G4) that was retrovirally transduced to express the CD155-specific A11 TCR reported previously (11) (Figure 3B). Indeed, responses of the three T-hybridomas and the A11 TCR-transduced 4G4 cell line to ligand-transfected 293T stimulator cells is blocked by soluble anti-CD3ε mAb, indicating that each response is transduced by CD3-dependent TCR components. We conclude that the reactivity of T-hybridomas is signaled by surface TCR complexes.

To determine the ligand binding affinity of one of these TCRs, we produced soluble TCR-146 and measured its binding to recombinant ICAM-2 (CD102) by surface plasmon resonance (SPR) in a completely cell-free assay (Figure 3C). The soluble TCR-146 binds to immobilized murine CD102 with dissociation constant K_D_ of 1.6 μM and 0.5 μM, derived from equilibrium and kinetic fittings, respectively. No detectable binding of TCR-146 was observed to immobilized human CD102 under the same condition (Figure 3C). The kinetic association and dissociation rates, k_on_ and k_off_, are 1.15 × 10^4^ (1/Ms) and 5.12 × 10^−3^ (1/s), respectively, for TCR-146/mCD102 binding (Table 1). Compared to the ligand binding affinity of conventional (MHC-restricted) αβTCRs, ligand binding by the MHC-independent TCR-146 displayed relatively high ligand binding affinity. In addition, both of their kinetic rate constants, especially k_off_, are substantially slower than those of conventional MHC-restricted TCR-ligand interactions. Although the k_on_ is 3–5 fold slower, the dissociation rate k_off_ for TCR146/mCD102 binding is 10–100 times slower than that of conventional MHC-restricted TCRs (22, 23). Similar slow on and off rates were also observed for ligand binding by two previously reported CD155-specific MHC-independent TCRs (A11 and B12A) (11) (see Table 1). Indeed, the slower on and off kinetic binding rate constant resemble many antibody-antigen interactions (24). Thus, MHC-independent TCR-146 binds with high affinity to an epitope on native murine ICAM-2 (CD102).

**Table 1:** Binding properties between MHC-independent TCRs(MHCi-TCR) and their ligands.

**MHCi-TCRs**	**Ligands**	**K_**D**_(μM)**	***k*_**off**_(s^**−1**^)**	***k*_**on**_(M^**−1**^s^**−1**^)**
TCR146	mICAM2	0.55 ± 0.25	5.12 ± 2.38 × 10^−3^	1.15 ± 0.95 × 10^4^
TCRA11	mCD155	0.26 ± 0.99	4.13 ± 0.91 × 10^−3^	1.72 ± 0.47 × 10^4^
TCRB12A	mCD155	0.23 ± 0.08	4.08 ± 1.34 × 10^−3^	1.96 ± 0.91 × 10^4^

### TCR-146 Recognizes ICAM-2 Protein Independently of LFA-1

**Figure 4:**
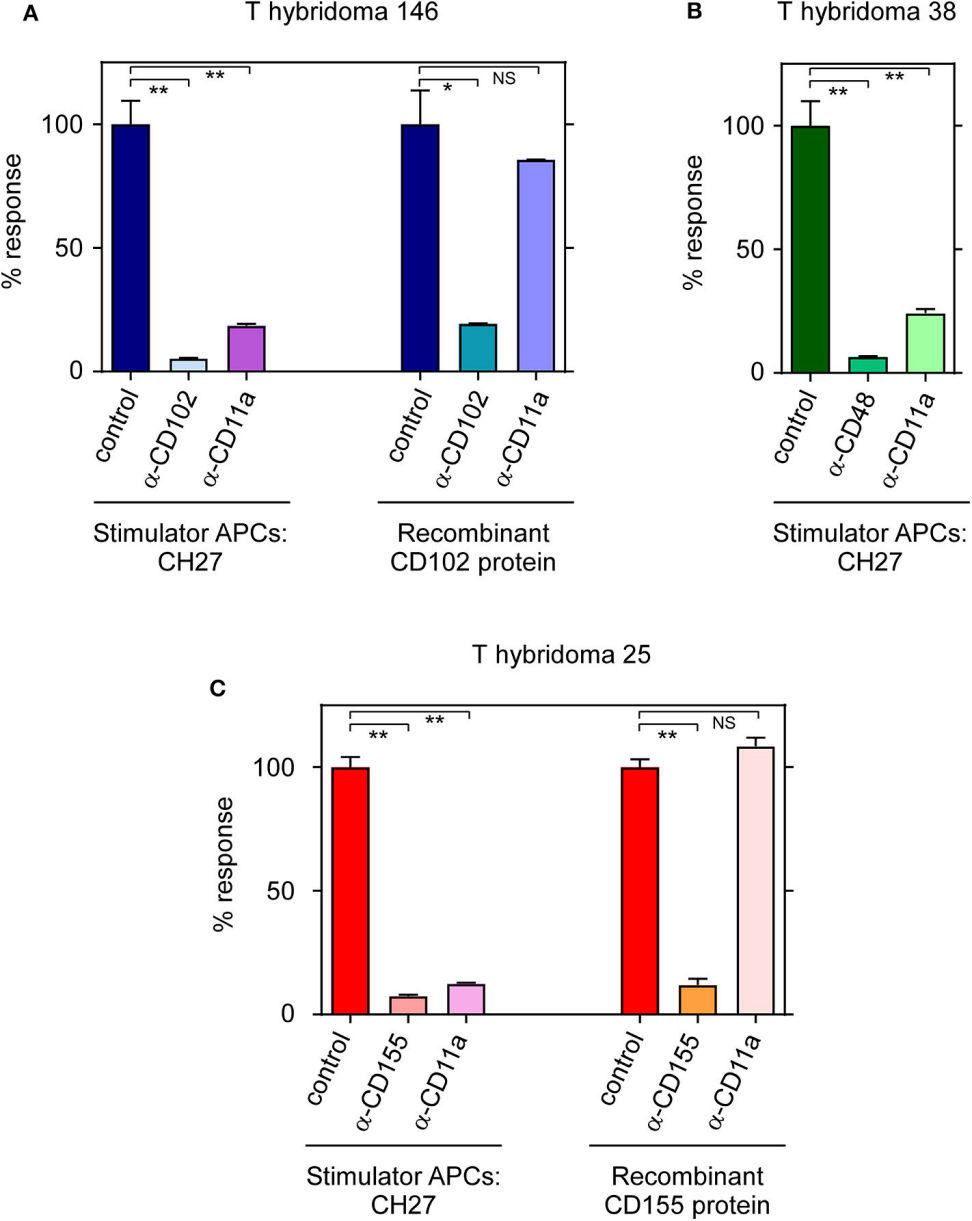
TCR-146 and TCR-25 require LFA-1-dependent interactions for cell-to-cell dependent stimulation. **(A)** Responses of T-hyb 146, **(B)** T-hyb 38, and **(C)** T-hyb 25 against CH27 stimulator cells or plate-bound recombinant proteins in the presence or absence of blocking antibodies. T-hyb responses in the absence of blocking antibodies were set at 100%. Each data point represents the mean ± SEM of triplicate experiments. **p < 0.01; *p < 0.05; NS, not significant.

In addition to being the stimulatory ligand for TCR-146, ICAM-2 (CD102) is also a low affinity ligand for the adhesion molecule LFA-1 which consists of a dimer composed of CD11a and CD18 chains on endothelial cells, monocytes, platelets and lymphocytes. The cellular adhesion molecule LFA-1 binds to several adhesive ligands (including ICAM-1, ICAM-2, and ICAM-3) (25–27) to promote cell-cell interactions which can be blocked by anti-LFA-1 mAb. We then assessed anti-LFA-1 blockade of each of the three T-hybridomas against their specific ligands (Figure 4). Interestingly, we found that anti-LFA-1 mAb non-specifically blocks all three T-hybridoma responses against cell-bound ligands but fails to block stimulation of these same T-hybridomas by plate-bound ligands–even when the plate-bound ligand is ICAM-2 (CD102) which is itself a ligand for LFA-1 (Figures 4A–C and Figure S4). Thus, LFA-1 promotes ligand-nonspecific adhesion between T-hybridomas and stimulator cells that is necessary for cell-bound TCRs to subsequently engage their cell-bound ligands, but LFA-1 is not required for TCRs to engage any cell-free ligands–including recombinant ICAM-2 (CD102) for which TCR-146 has higher binding affinity than LFA-1 (28).

### MHC-Independent Thymic Selection of TCR-146

**Figure 5:**
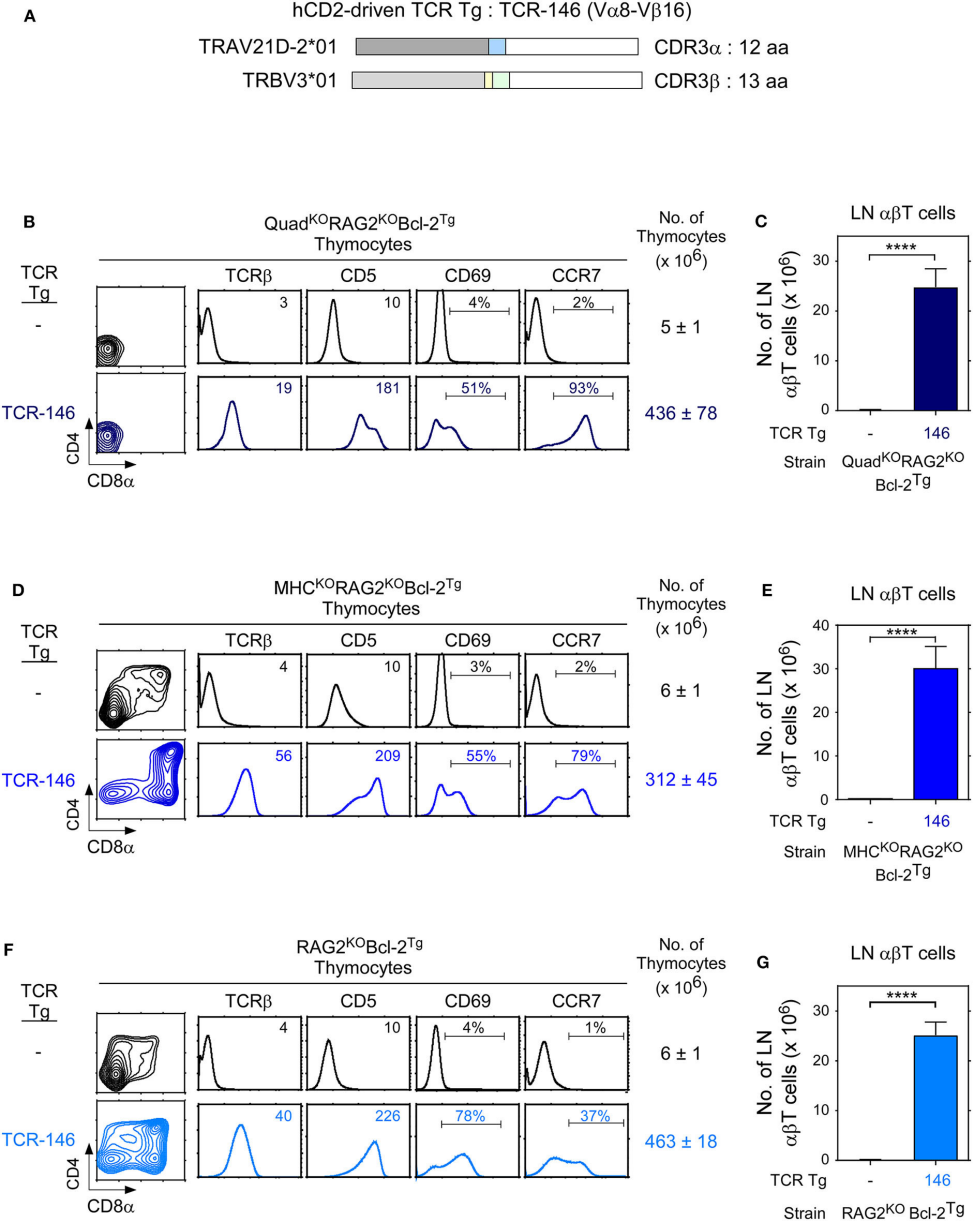
Thymic selection of transgenic TCR-146 requires neither MHC nor CD4/CD8 coreceptors. **(A)** hCD2-driven TCR-146 transgenic constructs encoding TCRα and TCRβ chains. **(B,D,F)** Thymocyte profiles from host transgenic mice expressing transgenic TCR-146. Numbers in TCRβ and CD5 histograms indicate Mean Fluorescence Intensity (MFI). Numbers in CD69 and CCR7 histograms indicate frequency of positive cells. Thymus cellularity is shown as mean ± SE (*n* = 3–9 mice/group). **(C,E,G)** Numbers of LN αβT cells in TCR transgenic mice (mean ± SE, *n* = 3–9 mice/group). ****p < 0.0001.

To examine positive selection in the thymus, we constructed a hCD2-driven transgene with TCR-146 (TCR-146^Tg^) that is specific for a novel MHC-independent ligand (Figure 5A) and we introduced TCR-146^Tg^ into Rag2^KO^Bcl-2^Tg^ host mice (Figures 5B–G). To assess if MHC and coreceptor expression, or lack thereof, affected TCR-146 signaling of positive selection, we systematically analyzed thymic selection signaling by TCR-146 in: (i) Quad^KO^Rag2^KO^Bcl-2^Tg^ host mice that were both MHC-deficient and coreceptor-deficient (Figures 5B,C), (ii) MHC^KO^Rag2^KO^Bcl-2^Tg^ host mice that were MHC-deficient but coreceptor-sufficient (Figures 5D,E), and (iii) Rag2^KO^Bcl-2^Tg^ host mice that were both MHC-sufficient and coreceptor-sufficient (Figures 5F,G).

TCR-146 effectively signals Quad^KO^Rag2^KO^Bcl-2^Tg^ thymocytes to undergo positive selection as revealed by thymocyte upregulation of CD5, CD69, and CCR7 and as revealed by generation of peripheral LNT cells (Figures 5B,C). Thus, unlike conventional MHC-restricted TCRs whose signaling of positive selection requires both MHC and CD4/CD8 coreceptor expression, TCR-146 signaling of positive selection requires neither MHC nor coreceptor expression.

Because hCD2-driven TCR transgenes are prematurely expressed in DN thymocytes before CD4/CD8 coreceptors are expressed, we thought that TCR-146 might access coreceptor-free Lck and signal MHC-independent positive selection in thymocytes at the DN stage of differentiation even in coreceptor-sufficient MHC^KO^ mice (12). In fact, TCR-146 did signal MHC^KO^Rag2^KO^ thymocytes to undergo positive selection and to generate large numbers of peripheral LNT cells (Figures 5D,E).

To assess TCR-146 signaling of positive selection in mice that are MHC-sufficient and coreceptor-sufficient, we introduced TCR-146^Tg^ into Rag2^KO^Bcl-2^Tg^ mice. We found that TCR-146 does signal Rag2^KO^Bcl-2^Tg^ thymocytes to undergo positive selection and to generate large numbers of peripheral LNT cells (Figures 5F,G), presumably because TCR-146 signaling occurs before thymocytes developmentally express coreceptor proteins on their cell surfaces. Taken together, all of the results in Figure 5 document that TCR-146 signaling of positive selection is coreceptor-independent and MHC-independent.

### ICAM-2 (CD102) Is the Required Thymic Selecting Ligand for TCR-146

**Figure 6:**
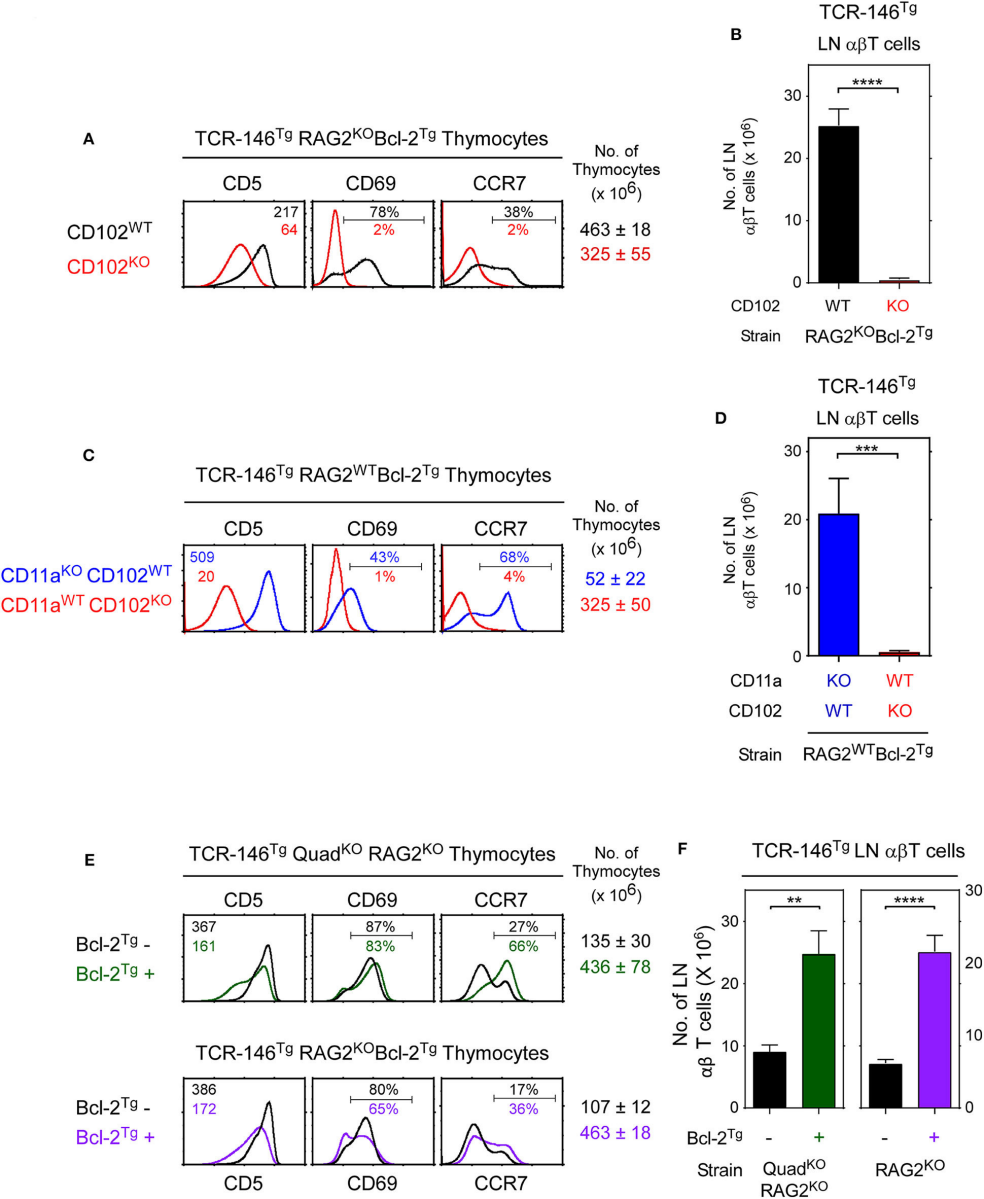
ICAM-2 (CD102) is the thymic selecting ligand for TCR-146. **(A)** *In vivo* positive selection signaled by TCR-146 requires ICAM-2 (CD102) expression. Numbers in CD5 histograms indicate MFI. Numbers in CD69 and CCR7 histograms indicate frequency of positive cells. Thymus cellularity is shown as mean ± SE of 6–10 mice/group. **(B)** Numbers of LN αβT cells in TCR transgenic mice (mean ± SE, *n* = 6–10 mice/group). **(C,D)** LFA-1 (CD11a expression) is not required for TCR146 signaling of positive selection *in vivo*. **(C)** Thymocyte profiles and **(D)** LN αβT cell numbers from transgenic mice represent the mean ± SE of 6–9 mice/group. **(E,F)** Comparison of thymocytes profiles and αβ LNT cell numbers in different Bcl-2^Tg+^ and Bcl-2^Tg−^ mouse strains expressing the TCR-146 transgene. ****p < 0.0001; ***p < 0.001; **p < 0.01.

We then considered if TCR-146 must encounter its high affinity ligand ICAM-2 to signal positive selection. To assess this possibility, we introduced TCR146^Tg^ into Rag2^KO^Bcl-2^Tg^ mice that are either CD102^WT^ and express ICAM-2, or are CD102^KO^ and lack ICAM-2 (Figure 6A). Importantly, we found that positive selection signaling by TCR-146 does strictly require ICAM-2, as TCR-146 does not signal positive selection and does not generate peripheral LNT cells in ICAM2-deficient CD102^KO^ mice (Figures 6A,B). Thus, TCR-146 strictly requires ICAM-2 to signal positive selection in the thymus.

However, it is possible that ICAM-2 might only promote ligand-non-specific LFA-1/ICAM-2 adhesive interactions needed for TCR-146 to engage other unknown positive selecting ligands in the thymus. Consequently, we compared TCR-146^Tg^ mediated positive selection in mice that are either LFA-1-deficient or ICAM-2-deficient (Figures 6C,D). We observed that TCR-146^Tg^ mediated positive selection is completely abrogated in ICAM-2-deficient (CD102^KO^) host mice but proceeds successfully in LFA-1-deficient (CD11a^KO^) host mice as determined both by thymocyte expression markers (CD5, CD69, CCR7) and by LNT cell generation (Figures 6C,D). Indeed, the number of TCR-146^Tg^ LNT cells in CD11a^KO^CD102^WT^ mice (~20–25 × 10^6^) is unaffected by LFA1-deficiency (compare CD102^WT^ mice in Figures 6B,D), even though LFA-1 deficiency non-specifically reduces LNT cell numbers in polyclonal CD11a^KO^ mice (Figure S5) (29). We conclude that ICAM-2 is the required positively selecting ligand for TCR-146 and that high affinity ligands are required for MHC-independent TCRs to signal positive selection in the thymus.

### TCR-146 Positive Selection Does Not Require Bcl-2^Tg^ Expression

We thought that TCR-146 engagement of its high affinity ligand ICAM-2 in the thymus might signal *in vivo* clonal deletion which was prevented in mice expressing the pro-survival Bcl-2^Tg^. Surprisingly, however, positively selected CCR7^+^ TCR-146 thymocytes appear in both Bcl-2^Tg+^ and Bcl-2^Tg−^ mice (Figure 6E) and differentiate into peripheral TCR-146 LNT cells in both Bcl-2^Tg+^ and Bcl-2^Tg−^ mice, albeit in lower (but still substantial) numbers in Bcl-2^Tg−^ mice (Figure 6F). Thus, despite engaging their high affinity ICAM-2 ligand in the thymus, many TCR-146 thymocytes survive thymic selection in Bcl-2^Tg−^ mice and differentiate into mature peripheral T cells, indicating that clonal deletion is incomplete (Figure 6F). We suggest that clonal deletion is incomplete because coreceptor-free Lck (which is the only Lck available to MHC-independent TCRs) transduces intracellular TCR signals so inefficiently that even high affinity TCR-ligand interactions fail to activate sufficient Lck to signal all TCR-146 thymocytes to undergo clonal deletion.

### The MHC-Independent TCR Repertoire Is Self-Reactive

**Figure 7:**
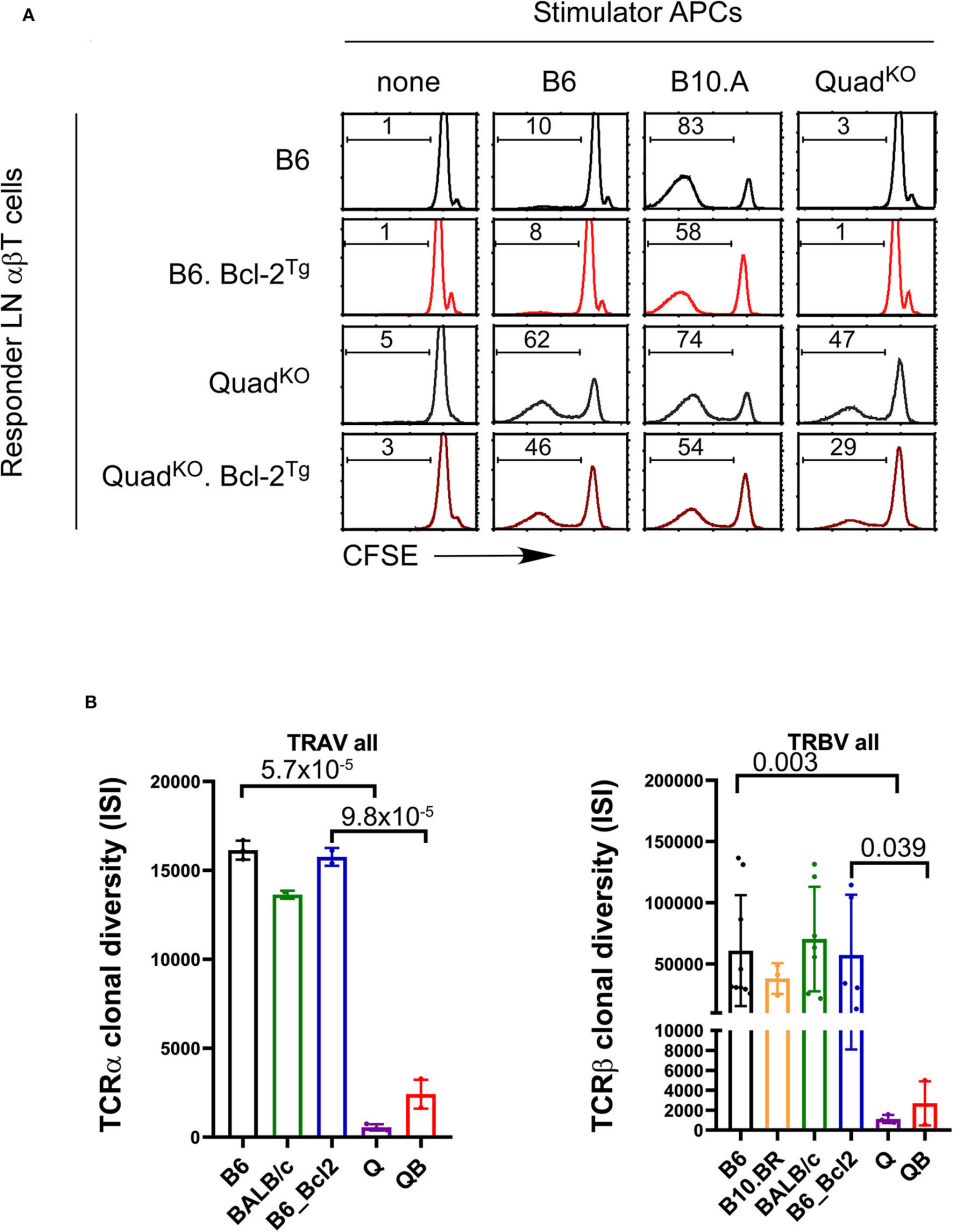
Increased TCR self-reactivity and diminished TCR repertoire diversity in polyclonal MHC-independent αβT cell populations. **(A)** T cell proliferative responses were measured by CFSE dye dilution of primary LN αβT cells from the indicated mice against various spleen cell stimulators. **(B)** TCR repertoire sequence diversities as measured by the Inverse Simpson Index of all TCRα sequences (left panel) and all TCRβ sequences (right panel) in the indicated mouse strains. *P*-values are shown of ISI diversity values between B6 and Quad^KO^ (Q) mice and between B6.Bcl-2^Tg^ and QB mice.

If coreceptor-free Lck is unable to efficiently signal clonal deletion, then self-reactivity might be a general feature of MHC-independent TCR repertoires in both Bcl-2^Tg+^ and Bcl-2^Tg−^ mice. To assess this possibility, we examined the reactivity of primary LNT cells from Bcl-2^Tg+^ and Bcl-2^Tg−^ mice against self and third-party spleen stimulator cells (T-depleted, LPS stimulated, irradiated spleen cells) in *in vitro* mixed lymphocyte responses as measured by CFSE dye dilution (Figure 7A). Interestingly, regardless of the presence or absence of Bcl-2^Tg^ expression, Quad^KO^ T cells are self-reactive as they react against syngeneic (Quad^KO^) stimulator cells as well as against third party B6 and B10.A spleen stimulator cells (Figure 7A). In contrast, neither B6 nor B6.Bcl-2^Tg^ T cells are self-reactive as they are unreactive against syngeneic (B6) spleen stimulator cells (Figure 7A). Thus, the MHC-independent TCR repertoire in Quad^KO^ mice is self-reactive in both the presence and absence of *in vivo* Bcl-2^Tg^, consistent with our concept that coreceptor-free Lck is too inefficient in transducing high affinity TCR signals to effectively delete autoreactive thymocytes and prevent their appearance in the periphery.

### Limited Diversity of the Peripheral Polyclonal MHC-Independent TCR Repertoire

Affinity is a measure of how well-receptor and ligand fit together, with low affinity indicating a poor fit and high affinity indicating a near-perfect fit. Because many different receptor structures would create a poor fit, many different TCR sequences might bind a ligand with low affinity; whereas few receptor structures would create a near-perfect fit and bind a ligand with high affinity. Similarly, low affinity positive selection would signal many different TCRs and generate a highly diverse receptor repertoire, whereas high affinity positive selection would signal few different TCRs and generate a receptor repertoire of limited diversity. This reasoning predicts that the receptor diversity of peripheral MHC-restricted TCRs would greatly exceed that of peripheral MHC-independent TCR repertoires.

To evaluate this prediction, we compared the diversity of TCRα and TCRβ sequence repertoires from the periphery of MHC-restricted B6, B10.BR, BALB/c, and B6.Bcl2 mice vs. TCRα and TCRβ sequence repertoires from the periphery of MHC-independent Quad^KO^ (Q) and QB mice as quantified by the Inverse Simpson Index (ISI) (Figure 7B and Figure S6). Note that the greater the ISI value, the greater the diversity of sequences within a TCR repertoire. Remarkably, we found that overall TCRα and TCRβ sequence diversities of peripheral MHC-independent TCRs are dramatically lower than those of MHC-restricted TCRs (Figure 7B and Figure S6). On average overall, the sequences of MHC-independent TCR repertoires are 10–50 fold less diverse that those of MHC-restricted TCRs. Figure 7B summarizes overall TCRα and TCRβ clonal diversity in each mouse strain examined, without accounting for variations in V-gene usage. Figure S6 displays TCRα and TCRβ clonal diversity among each individual V-gene in the mouse strains examined, and reveals that the repertoire diversity of MHC-restricted TCRs in B6 mice is far greater than the repertoire diversity of MHC-independent TCRs in Quad^KO^ mice (*p* < 10^−15^), and the repertoire diversity of MHC-restricted TCR in B6.Bcl-2^Tg^ mice is far greater than the repertoire diversity of MHC-independent TCRs in QB mice (*p* < 10^−12^ to 10^−15^). Based on these findings, we conclude that positive selection by high affinity TCR-ligand engagements severely reduces TCR repertoire diversity and increases self-reactivity.

## Discussion

The present study reveals that MHC-independent TCRs require high affinity TCR-ligand engagements to signal positive selection and that high affinity positive selection generates a mature repertoire with increased self-reactivity and markedly reduced TCR diversity. In this study we first identified novel MHC-independent TCRs in Quad^KO^ mice that were reactive against three native self-proteins which otherwise functioned as low affinity cell adhesion molecules. We focused on TCR-146 which was reactive against ICAM-2 (CD102), a low affinity ligand for the cell adhesion molecule LFA-1, and which bound soluble ICAM-2 (CD102) independently of LFA-1 with relatively high 1.6 μM affinity. To assess *in vivo* positive selection signaling by TCR-146, we constructed TCR-146 transgenic mice and found that TCR-146 strictly required its high affinity ligand ICAM-2 to signal positive selection in the thymus, demonstrating that high affinity positive selection signaling was not unique to CD155-specific TCRs (12) but was in fact a general feature of MHC-independent TCRs. Moreover, we discovered that high affinity positive selection signaling in Quad^KO^ mice selected polyclonal TCRs that were self-reactive and, most surprisingly, with very limited repertoire diversity. Thus, this study indicates that the affinity of TCR-ligand engagements required to signal positive selection in the thymus affects, inversely, the diversity and self-tolerance of the mature TCR repertoire.

The requirement for high affinity TCR-ligand engagements to signal positive selection is unique to MHC-independent TCRs as MHC-restricted TCRs signal positive selection by engaging very low affinity ligands. The obvious difference in TCR-ligand affinities required to signal positive selection of immature thymocytes is quite surprising because positive selection requires uniquely weak TCR signals–and this should be the case regardless of ligand specificity. While TCR signal strength is often equated with TCR-ligand affinity, this study requires that TCR signal strength and TCR affinity be conceptually separated. When this is done, the positive selection requirement for weak TCR signaling means that only few activated p56^Lck^ (Lck) tyrosine kinase molecules must be recruited to ligand-engaged surface TCR complexes. So the different TCR affinities required by MHC-restricted and MHC-independent TCRs to signal positive selection reflect the different efficiencies with which Lck is recruited to different ligand-engaged TCR complexes. In CD4/CD8 coreceptor-sufficient mice, Lck is associated with the cytosolic tails of CD4/CD8 coreceptors that bind to surface pMHC ligands together with MHC-restricted TCRs, so that coreceptors efficiently bring Lck to ligand-engaged TCRs. In contrast, in CD4/CD8 coreceptor-deficient mice, Lck is coreceptor-free and must be passively captured within surface TCR clusters, a process which is highly inefficient and requires high affinity TCR-ligand engagements that persist long enough to passively capture free Lck within TCR-ligand clusters. Thus, the weak TCR signals that induce positive selection are generated either by low affinity coreceptor-dependent TCR-ligand engagements or by high affinity coreceptor-independent TCR-ligand engagements, with the former generating an MHC-restricted TCR repertoire and the latter generating an MHC-independent TCR repertoire.

The requirement for high affinity TCR-ligand engagements to signal positive selection profoundly effects the self-reactivity and diversity of the mature TCR repertoire. Regarding the self-reactivity of the mature TCR repertoire, high affinity positive selection necessarily generates a peripheral MHC-independent TCR repertoire with high affinity for self-protein ligands. Even so, it is surprising that MHC-independent TCRs are incompletely self-tolerant to their own self-proteins as revealed by their self-reactivity in mixed lymphocyte cultures *in vitro*. Indeed, their self-reactivity is not limited to *in vitro* assays as MHC-independent Quad^KO^ mice have *in vivo* lymphocytic infiltrations into multiple organs beginning at ~6 months of age (10). As explanation, we think that MHC-independent TCRs with high affinity for self-ligands are not fully deleted in the Quad^KO^ thymus because free Lck is so inefficient at transducing TCR signals that high affinity TCR-ligand interactions fail to signal strongly enough to induce clonal deletion. While high affinity MHC-independent TCRs that avoided clonal deletion in Quad^KO^ mice might have been expected to become T-regulatory cells, we found that T-regulatory cells account for only ~5% of peripheral T cells in Quad^KO^ mice (unpublished). Instead, we think that peripheral MHC-independent T cells become self-reactive in the periphery because mature T cells increase their intra-cellular content of Lck during their differentiation from immature thymocytes which generates stronger TCR signals in the periphery.

Failure of high affinity MHC-independent TCRs to induce clonal deletion can provide insight into how much CD4/CD8 co-receptors contribute to the strength of intracellular signal transduction during thymic selection. In our thinking negative selection in the thymus requires that sufficient Lck be recruited to ligand-engaged TCR complexes to transduce strong intracellular signals that induce immature thymocyte death. In the absence of CD4/CD8 coreceptors, recruitment of coreceptor-independent Lck to ligand-engaged TCRs is inefficient and requires long duration TCR binding to the negatively selecting ligand as best measured by dwell time (30). In contrast, CD4/CD8 coreceptors efficiently bring Lck to ligand-engaged TCRs which consequently require much shorter duration TCR binding to the negatively selecting ligand. In fact, the dwell time of the high affinity OT-I MHC class I-restricted TCR on its negatively selecting pMHC ligand was determined to be ~ 0.2–1 s (30), with similar dwell times subsequently determined for other MHC-restricted TCRs (23). In marked contrast to the short ligand dwell times of MHC-restricted TCRs, the dwell time of MHC-independent TCR-146 on its thymic selecting ligand ICAM-2 is ~130 s which is 100–200 times greater, with similarly long dwell times of 168–170 s for the binding of two previously reported MHC-independent TCRs, A11 and B12A (11, 31), to their CD155 ligand. We suggest that the much shorter dwell times required of MHC-restricted than MHC-independent TCRs reveal the major contribution of CD4/CD8 coreceptors to the strength of intracellular signal transductions required for clonal deletion in the thymus. Notably, while a few MHC-restricted TCRs on peripheral T cells can signal independently of CD4/CD8 coreceptors, that is not true for thymic selection of those same TCRs which is strictly coreceptor-dependent in the thymus because Lck in immature DP thymocytes is all coreceptor-bound (32–34). Moreover, concordant with our concept that TCR-specific clonal deletion cannot be efficiently transduced in thymocytes by coreceptor-free Lck, we previously showed that T cells bearing self-reactive MHC-independent TCRs were not deleted in mice whose thymocytes normally expressed MHC and CD4/CD8 coreceptors but contained re-engineered coreceptor-free Lck that was unable to bind to CD4/CD8 coreceptors (12).

Regarding the limited repertoire diversity of MHC-independent TCRs, we think limited diversity is a necessary outcome of the positive selection requirement for high affinity TCR engagements. Because affinity is a measure of how well-receptor and ligand fit together with high affinity indicating a near-perfect fit, a high affinity requirement for positive selection limits positive selection signaling to only the few MHC-independent TCRs able to bind an individual ligand with sufficient affinity to signal positive selection. In contrast, a low affinity positive selection requirement allows many different poorly fitting TCRs to engage an individual ligand and signal positive selection. As a result, a high affinity positive selection requirement severely limits the number of different TCRs that can be selected into the mature repertoire by an individual self-ligand in the thymus, whereas a low affinity positive selection requirement allows multiple, even millions, of different TCRs to be selected into the mature repertoire by an individual self-ligand in the thymus (35). An important implication of this reasoning concerns TCR reactivity to foreign ligands. Because all peripheral TCRs are originally selected by self-ligands in the thymus, TCR recognition of foreign ligands is entirely the result of fortuitous cross-reactivities. Consequently, peripheral TCR repertoires with limited diversity are far less likely to fortuitously recognize and react against a foreign pathogenic ligand. Consequently, the peripheral MHC-independent TCR repertoire is likely to be deficient in recognizing and reacting to foreign pathogenic ligands.

Based on this study, we suggest that MHC-restricted TCRs with low affinity positive selection requirements arose as a result of evolutionary pressure to generate a maximally diverse and, therefore maximally protective, TCR repertoire. Selection of a highly diverse and more self-tolerant TCR repertoire was achieved during evolution by employing CD4/CD8 coreceptors with cytosolic tails that bound Lck and extracellular domains that bound to MHC, since CD4/CD8 coreceptors efficiently recruited Lck to MHC-engaged TCRs which markedly lowered the affinity of TCR-ligand engagements required to signal positive selection in the thymus. Notably, since CD4 and CD8 coreceptor external domains only bind to different classes of MHC proteins, the involvement of CD4 and CD8 coreceptors in thymic selection necessarily resulted in low affinity positive selection of a peripheral TCR repertoire that was MHC-restricted, highly diverse, and self-tolerant.

It is a curious feature of the MHC-independent TCR repertoire that the ligands identified so far are involved in cell adhesion. We suspect this is because cell adhesion ligands are highly expressed on cells in the thymus which is necessary for sufficient numbers of TCR-ligand engagements to form and signal positive selection. In addition, we previously noted that cell adhesion molecules like CD155 are downregulated during T-hybridoma fusions which decreases the fratricide of T-hybridomas that bear TCRs with those ligand specificities and increases their relative recovery in T-hybridoma fusions (36).

Finally, naturally arising MHC-independent TCRs generated by Rag-mediated gene rearrangements resemble re-engineered therapeutic CAR T cells in recognizing self-ligands independently of MHC (37, 38). They differ from re-engineered CARs in consisting of normal αβTCR chains which transduce signals in the same way as conventional αβTCRs and differently than CAR T cells. Consequently, we think naturally arising MHC-independent αβTCRs with MHC-independent specificity for tumor antigens may possibly prove to be of greater therapeutic usefulness than CARs.

In conclusion, this study provides novel insights into the relationship between the required affinity of TCR-ligand engagements that signal positive selection in the thymus and critical features of the mature TCR repertoire in the periphery. This study suggests the novel concept that the required affinity of positive selection signaling in the thymus determines, inversely, the diversity and self-tolerance of the peripheral TCR repertoire.

## Materials and Methods

### Animals

MHC-deficient (*B2m*^−/−^*H-2-Ab1*^−/−^), RAG-deficient (*Rag2*^−/−^), Quad-deficient (*B2m*^−/−^*H-2-Ab1*^−/−^*Cd4*^−/−^*Cd8a*^−/−^) (10), and Quad-deficient.Bcl2 mice containing the hBcl2 transgene (39) were bred in our own animal colony. Mice deficient in CD155 (*Cd155*^−/−^) were generated as previously described (21), as were ICAM-2 (*Cd102*^−/−^) deficient mice (40) and LFA-1 (*Cd11a*^−/−^) (18). Animal care was in accordance with National Institutes of Health (NIH) guidelines.

New transgenic mouse strains constructed for this study were generated by cloning full length TCR cDNAs for TCRα and TCRβ into the human CD2 transgenic vector to obtain T cell specific expression.

### Generation of T Cell Hybridomas

Lymph node αβT (LNT) cells from Quad^KO^ and Quad^KO^Bcl-2^Tg^ (QB) mice were stimulated with plate-bound anti-TCRβ/CD28 (5 and 2 μg/ml) for 48 hr. fused to TCR-null BW5147 cells, and subcloned at <1 cell/well (10). IL-2 secretion was measured by enzyme-linked immunosorbent assays (ELISA) (R&D Systems) after overnight stimulation. Hybridomas were screened for TCR reactivity (using plate-bound anti-TCR antibodies) as well as reactivity against LPS activated- MHC- and CD155-deficient splenic B cells and the murine B lymphoma CH27 cells (41).

### Antibodies and Reagents

MAbs with the following specificities were used in the present study: CD4 (GK1.5 or RM4.4), TCRβ (H57-957), CD5 (53-7.3), CD8α (53-6.7), CD69 (H1.2F3), CCR7 (4B12) were obtained from BD Biosciences (San Jose, CA). LEAF-purified antibodies against mouse CD155 (clone 4.24.1) were obtained from Biolegend. Functional grade antibodies against mouse CD102 (clone 3C4 (mIC2/4), mouse CD54 (clone YN1/1.7.4), mouse CD11a (LFA-1 alpha, clone M17/4) were obtained from eBiosciences, mouse CD48 (clone HM48-1) was obtained from Biolegend. Purified anti-CD3 antibodies [clone 7D6 (42)] were generated in house.

### Stimulation With Plate-Bound Ligands

Flat-bottom 96-well plates were coated overnight with recombinant proteins in 50 μl of PBS. Hybridomas were added overnight, after which supernatants were assessed for IL-2.

### Construction and Screening of the cDNA Library

Total RNA was prepared from the murine CH27 cell line by RNeasy Maxi (QIAGEN Inc.) and was purified with FastTrack MAG Maxi mRNA isolation kit (Invitrogen) to obtain poly(A)+ RNA. cDNA was synthesized with the SuperScript system (Invitrogen) and was cloned into SPORT6 vector with SalI and NotI restriction sites. ElectroMAX DH10B competent cells (Invitrogen) were transformed by electroporation, and after titration, *E. coli* (~150 clones/well) were inoculated overnight into 96-well format culture blocks (10 blocks). Plasmids were purified with a Qiaprep 96 Turbo miniprep kit (QIAGEN) and were transfected to HEK293T cells with Lipofectamine 2000 (Invitrogen) in 96-well flat-bottom plates and left overnight. Hybridomas were cocultured with cDNA-transfected 293T cells for 24 h, after which mIL-2 amounts in the supernatants were obtained. Positive clones were selected for secondary and tertiary screenings. Subpool libraries (~20 clones/well, 48 wells) and clone libraries (1 clone/well, 96 wells) were prepared and screened. Positive clones were sequenced to identify the specificity of the transfected cDNA.

### Flow Cytometry

Cells from thymi and lymph nodes were analyzed on an LSRII (BD Biosciences) and dead cells were excluded by forward light-scatter and propidium-iodide uptake.

### Generation of Soluble αβTCR Heterodimers

DNA encoding extracellular portions of TCR α and β chains were cloned into pET30a vectors as described (11). Receptors were expressed as inclusion bodies in BL21 (DE3) cells. Functional and soluble TCR heterodimers were produced by a rapid dilution refolding procedure as previously described (43). Refolded TCR heterodimers were purified using anion exchange chromatography followed by size exclusion chromatography.

### Surface Plasmon Resonance

Surface plasmon resonance measurements were performed using a BIAcore 3000 instrument and analyzed with BIAevaluation 4.1 software (Biacore AB). Murine ICAM-2-Fc, human ICAM-2-Fc and NKp44-Fc fusion proteins (R&D systems) were immobilized on carboxylated dextran CM3 chips (Biacore AB) to 4000–7000 response units (RU) using a primary amine-coupling in 10 mM sodium acetate (pH 5.0). The analytes consisted of serial dilutions of TCR-146 between 8 and 0.5 μM in a buffer containing 10 mM Hepes (pH7.4), 0.15 M NaCl, 1 mM CaCl2 and 0.5 mg/ml BSA. The dissociation constants were obtained by kinetic curve fitting for the binding of TCR146 to murine ICAM-2 using BIAevaluation 4.1 (BIAcore Inc.).

### T Cell Proliferation

To test T cell reactivity against stimulator cells from different mouse strains, we labeled purified LNT responder cells with CFSE and cocultured them with irradiated (2000 rads) LPS-activated splenic B cell blasts. Cultures were analyzed on day 4 by multicolor flow cytometry.

### Epitope Mapping

Chimeric CD155 molecules were generated as described (11). Briefly, chimeric CD155 molecules between mouse and human were generated. The chimeric constructs were synthesized (GenScript) and cloned into pIRES2-ZsGeen1 expression vector (Clontech). Hybridoma cells were cocultured with 293T cells transfected with chimeric cDNA for 24 hr after which supernatant IL-2 was measured.

### Analysis of T Cell Repertoire Sequence Diversity

TCRα and β repertoire sequences for all indicated mouse strains were obtained by high throughput next generation RNA sequencing through Adaptive Biotechnologies Corp. and iRepertoire, Inc. Three or more animals from each strain were sequenced. Sequence diversity was estimated for each overall repertoire and for each individual Vα- and Vβ-gene family using Inverse Simpson Index (ISI) as calculated by AAfrequency, an in-house developed program (8).

## Data Availability Statement

TCRβ sequences used for diversity analysis are available through Adaptive Biotechnology's ImmuneAccess database [https://clients.adaptivebiotech.com/pub/lu-2019-natcomms] with doi: 10.21417/JL2019. Both TCRα and TCRβ sequences are also available upon request.

## Ethics Statement

The animal study was reviewed and approved by NCI Animal Care and Use Committee.

## Author Contributions

FV, IS, PS, and AS conceptualized and designed the study and interpreted the results. FV performed the experiments, analyzed data, and drafted the manuscript. IS, JL, AB, XT, TG, AA, MR, and JA performed experiments and contributed to the analysis of the data. KH, JY, and PS provided conceptual and technical support. BE provided an experimental animal model. AS conceived and supervised the research program and experiments, and wrote the manuscript. All authors listed have approved the work for publication.

## Conflict of Interest

The authors declare that the research was conducted in the absence of any commercial or financial relationships that could be construed as a potential conflict of interest.
