# Supplementary figures and images for: Novel MHC-Independent αβTCRs Specific for CD48, CD102, and CD155 Self-Proteins and Their Selection in the Thymus
Source: Front Immunol. 2020 Jun 16;11:1216. doi: 10.3389/fimmu.2020.01216 (PMC7308553; doi:10.3389/fimmu.2020.01216)

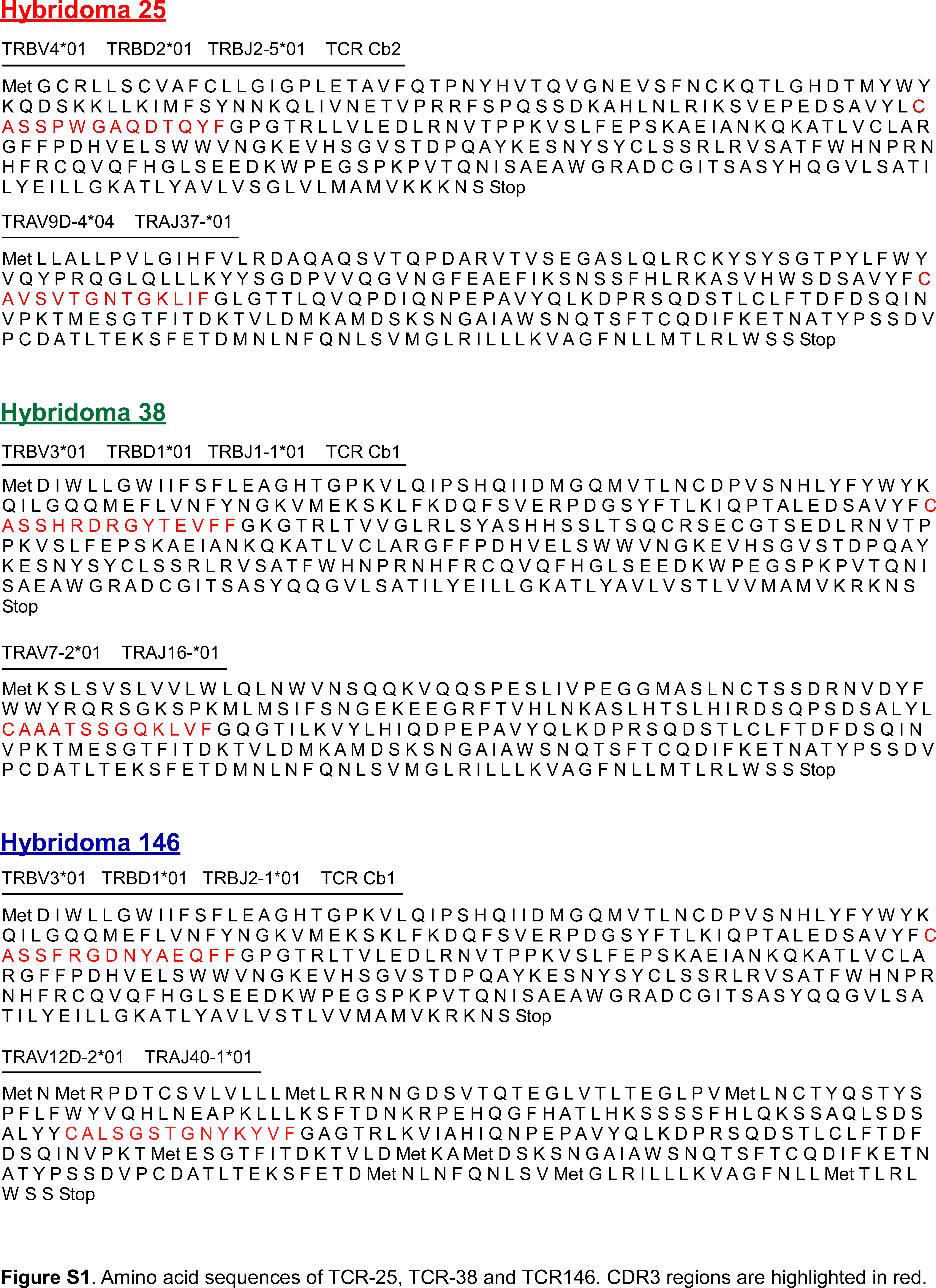

Supplement: Supplementary file 1 [file Image_1.TIF]

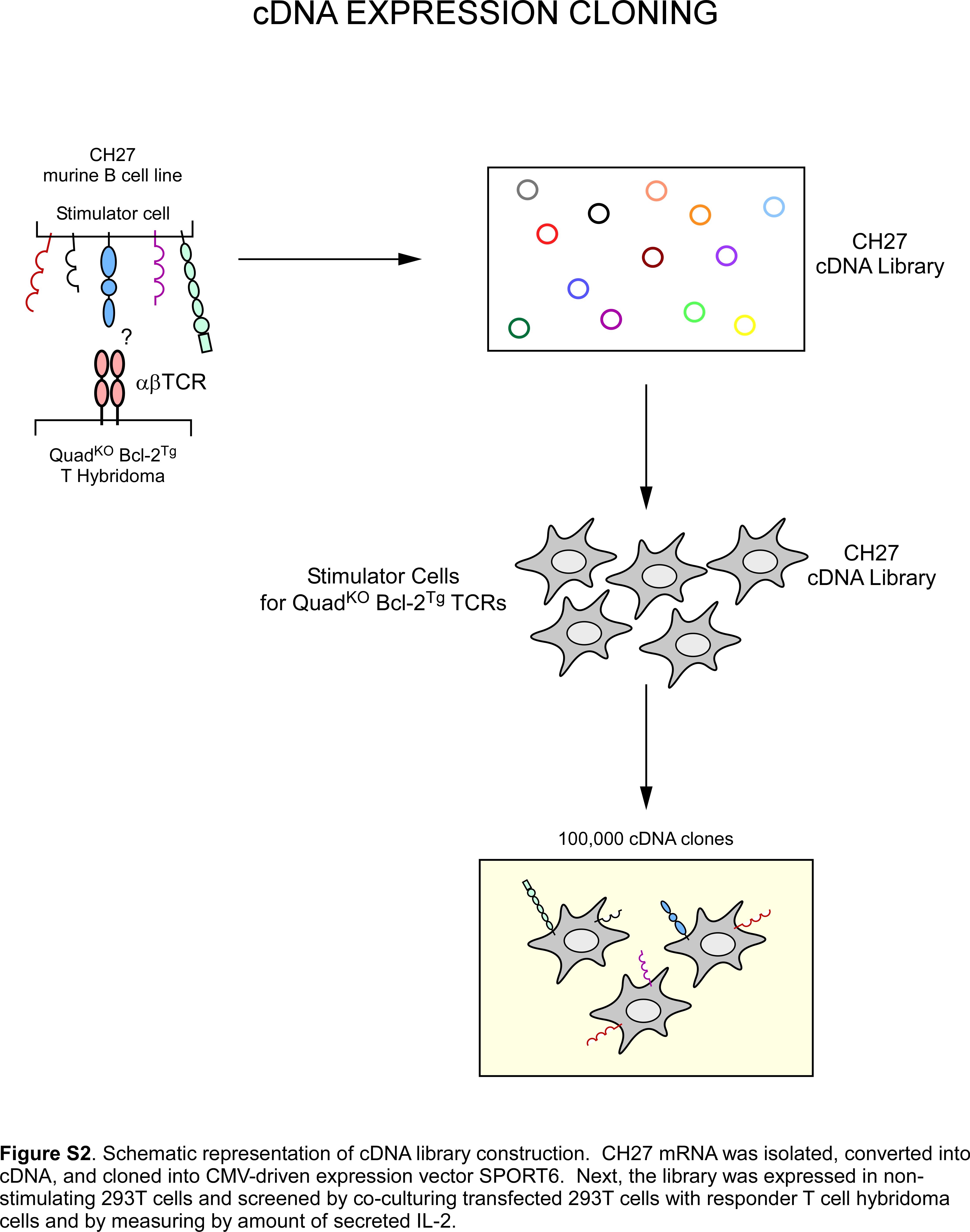

Supplement: Supplementary file 2 [file Image_2.TIF]

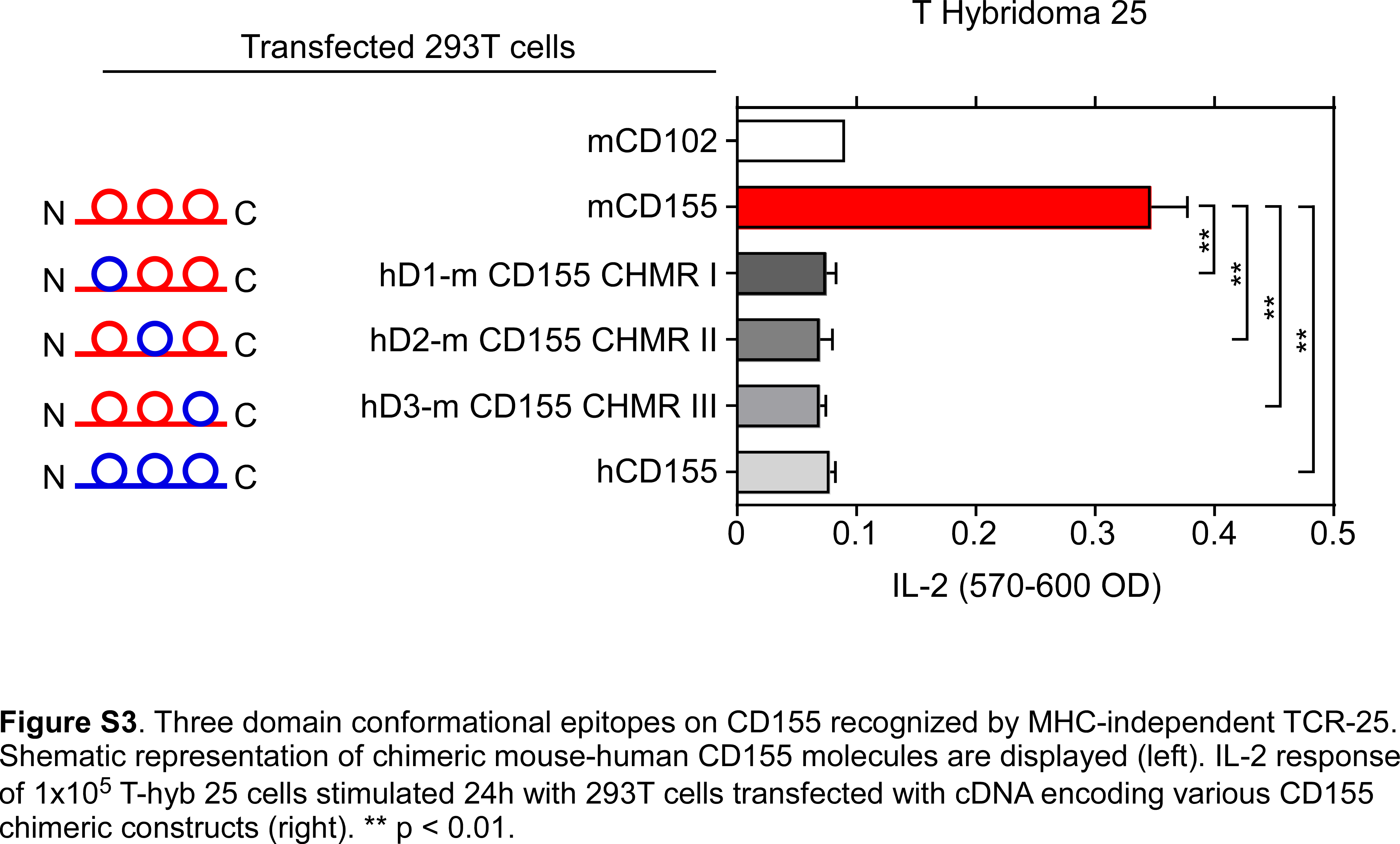

Supplement: Supplementary file 3 [file Image_3.TIF]

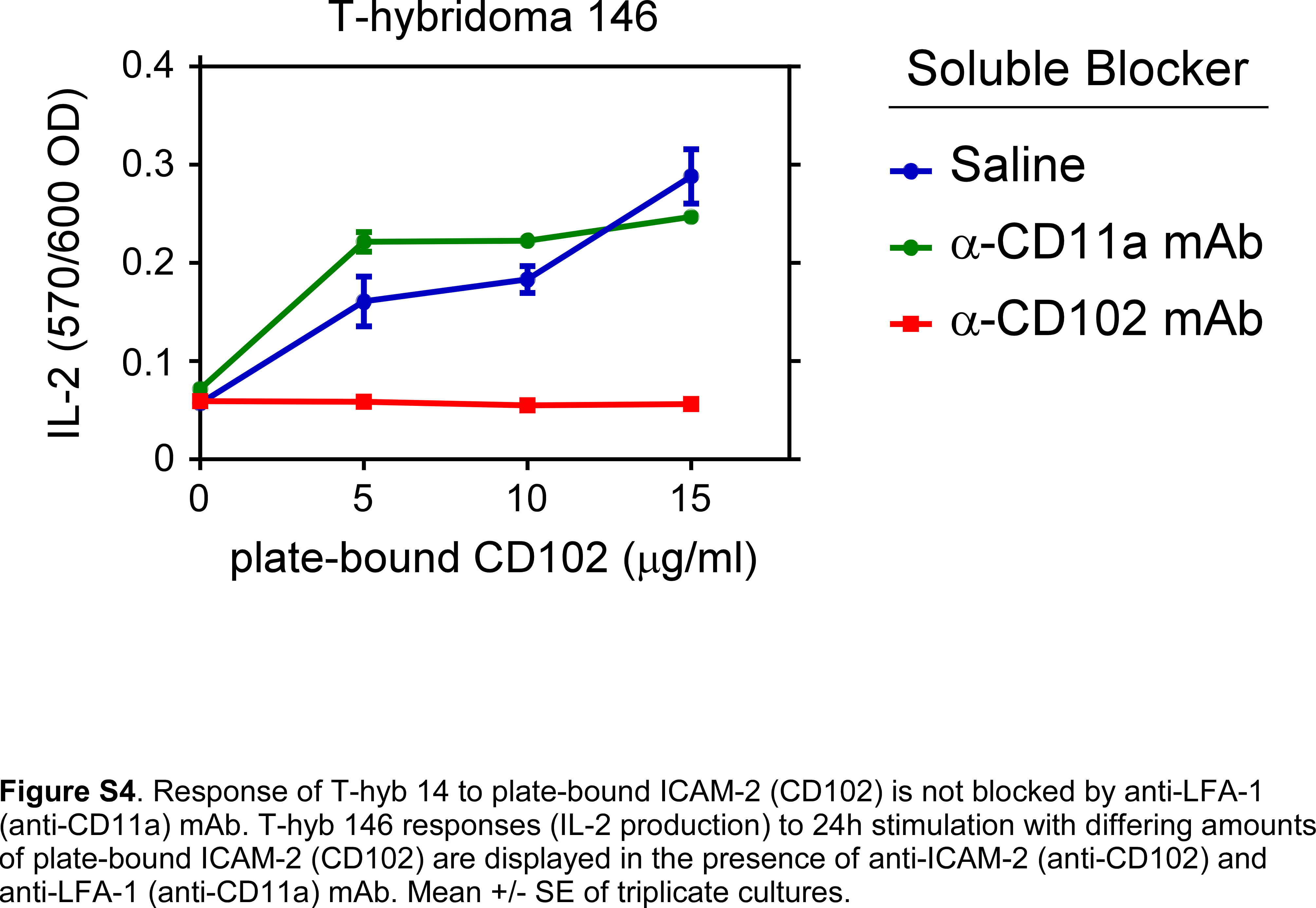

Supplement: Supplementary file 4 [file Image_4.TIF]

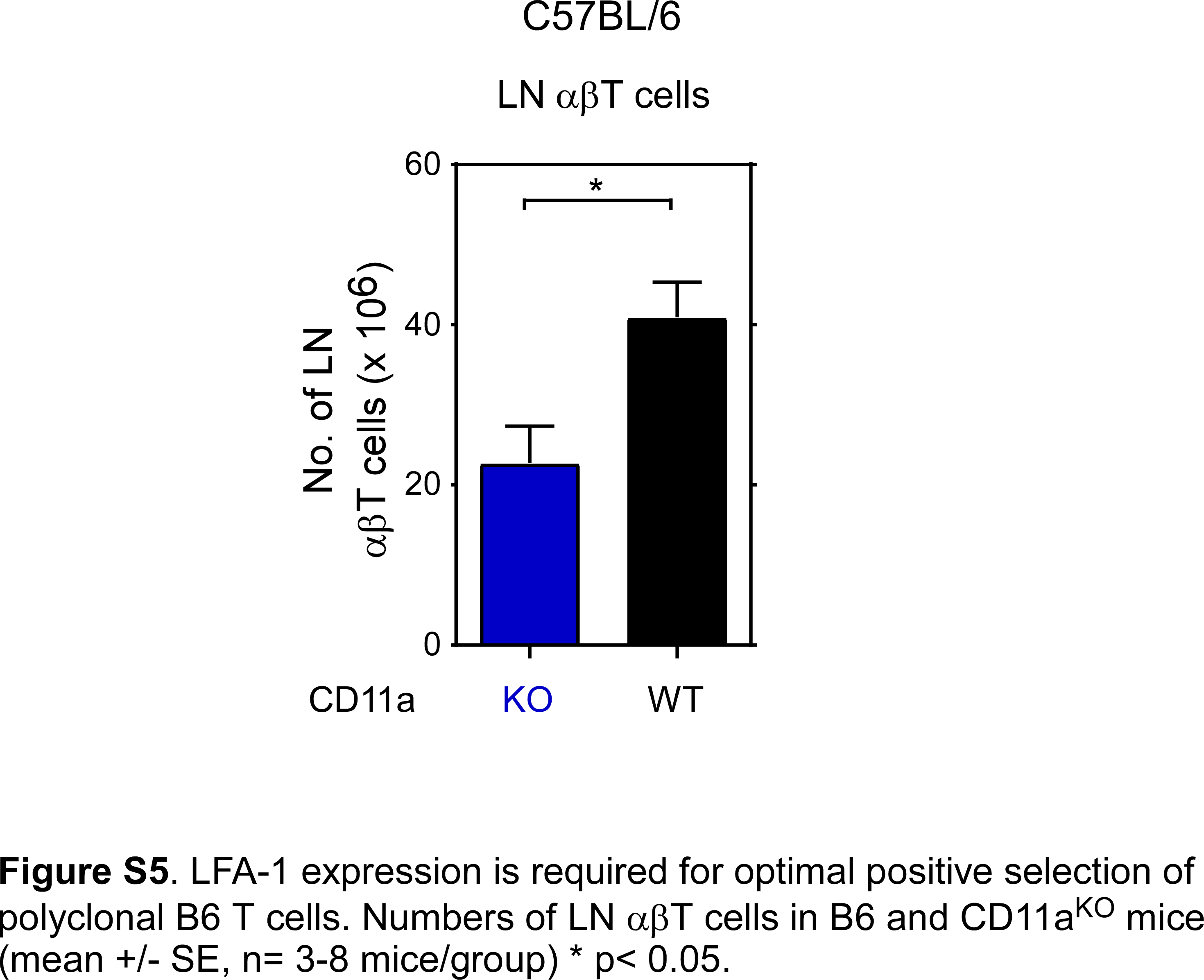

Supplement: Supplementary file 5 [file Image_5.TIF]

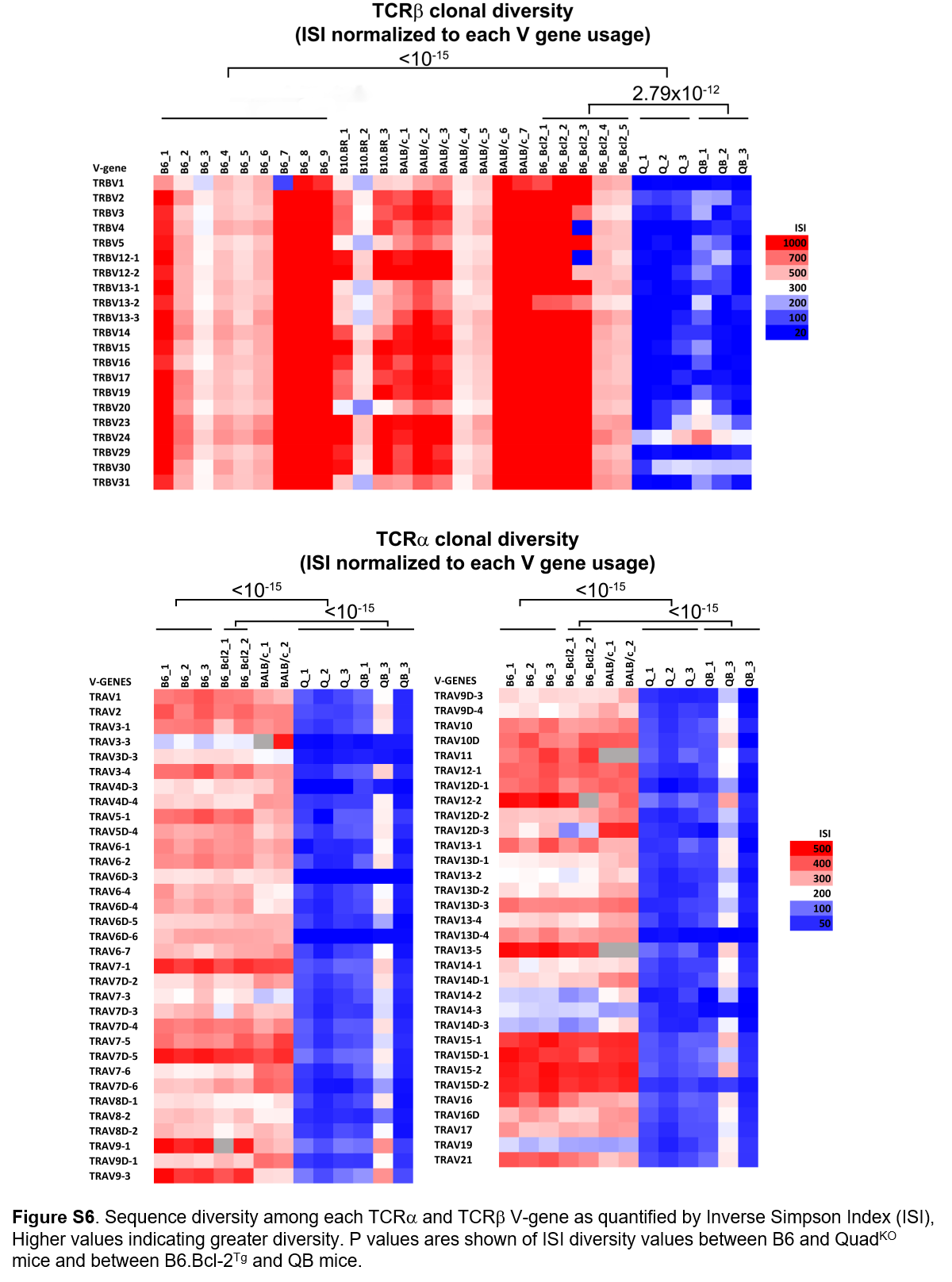

Supplement: Supplementary file 6 [file Image_6.TIF]
